# Dislocation Topological Evolution and Energy Analysis in Misfit Hardening of Spherical Precipitate by the Parametric Dislocation Dynamics Simulation

**DOI:** 10.3390/ma14216368

**Published:** 2021-10-25

**Authors:** Haiwei Zheng, Jianbin Liu, Shinji Muraishi

**Affiliations:** Department of Materials Science and Engineering, Tokyo Institute of Technology, Tokyo 152-8550, Japan; zheng.h.ac@m.titech.ac.jp (H.Z.); liujianbin202103@163.com (J.L.)

**Keywords:** simulation, the parametric dislocation dynamics, aluminum

## Abstract

Interaction of a single dislocation line and a misfit spherical precipitate has been simulated by the Parametric Dislocation Dynamics (PDD) method in this research. The internal stress inside the precipitate is deduced from Eshelby’s inclusion theory, the stress of the dislocation line and outside the precipitate is calculated by Green’s function. The influence of different relative heights of the primary slip plane on dislocation evolution is investigated, while the cross-slip mechanism and annihilation reaction are considered. The simulation results show three kinds of dislocation topological evolution: loop-forming (Orowan loop or prismatic loop), helix-forming, and gradual unpinning. The dislocation nodal force and the velocity vectors are visualized to study dislocation motion tendency. According to the stress–strain curve and the energy curves associated with the dislocation motion, the pinning stress level is strongly influenced by the topological change of dislocation as well as the relative heights of the primary slip plane.

## 1. Introduction

Interaction between the internal stress caused by the misfit precipitate and the dislocation plays an important role in metal solids’ strengthening. The advent of the Dislocation Dynamics (DD) allows researchers to simulate the precipitate-dislocation interaction in mesoscale and filling the gap between the atomistic simulation and the crystal plasticity theory [1].

In the 1960s, Brown [2], Bacon [3], and Foreman [4] proposed the methodology of the Discrete Dislocation Dynamics (DDD) simulation. They managed to get the curvature of the dislocation segments under the applied stress. In 1992, Kubin et al. [5] developed the first 3D discrete dislocation simulation tool, they assumed that the discrete edge-screw dislocation segments move on a discrete lattice superimposed to the crystallographic lattice. Zbib et al. [6] discretized dislocation segments as the linear splines to make the dislocation line smooth and flexible. Rhee et al. [7] managed to simulate the dislocation stress field in the anisotropy media. In 1998, they built a 3D dislocation dynamics model of the dislocation interactions including annihilation and the cross-slip based on a ‘critical-force’ criterion [8]. Zbib et al. [9] investigated the size-dependent small-scale plasticity phenomena in a multiscale framework, in nano-microscale the plasticity is determined by the explicit 3D dislocation dynamics, while in continuum scale the energy transport is based on the continuum mechanics laws. In 2008, Takahashi et al. [10] proposed a method combining the Parametric Dislocation Dynamics (PDD) and the Boundary Element Method (BEM) with the volume integrals. This method can be used to calculate the stress field both inside and outside the precipitate which has the different elastic modulus with the matrix. In 2018, Keyhani et al. [11] firstly attempted to systematically quantify the dislocation-precipitate interaction in terms of the applied shear stress, the precipitate resistance, and the required time to reach the critical state when a dislocation line is about to pass through the precipitate.

The present study is based on our former works done by Muraishi and Liu, who developed the PDD codes through Green’s function method. Before this, Muraishi and Liu have researched the dislocation interacts with an ellipsoidal precipitate in Al-Cu alloys [12], and the influence of the key parameters (existence of the cross-slip, the radius of precipitate and the dislocation source length) on the precipitate-dislocation hardening [13]. In this study, we mainly investigated the dislocation topological evolution, not only on the z/R≥0 planes but also the z/R<0 planes, which were considered in our previous study. The stress and energy curves are also plotted to explain the strengthening mechanism. The stress field inside the misfit precipitate with eigenstrain is computed through Eshelby’s inclusion theory [14,15]. The internal stress outside the precipitate, as well as the stress of the dislocation, is obtained from the integral form of elastic Green’s function. The dislocation structure information is stored in the linked list. The linked list data structure has advantages in representing the addition, deletion, break, and connection of the dislocation nodes by simply updating the pointers [16]. Therefore, it is convenient to simulate the dislocation reactions including the emission and annihilation through a linked list. The cross-slip which has a great impact on the dislocation bypassing mechanism, is also considered in this model. The dislocation nodal force and the velocity vectors are visualized for a deeper understanding of the dislocation motion tendency. Basically, after the dislocation line evenly swept the area of the positive and negative misfit stress, the interaction energy between the dislocation and the precipitate vanishes as a result. However, the dislocation line with two pinned endpoints cannot sweep such an area. Therefore, the hardening caused by the misfit stress is expected to be the pinning stress. Since the misfit stress is geometrical dependent, the cutting and bypassing behaviors are also shape-dependent [17]. Strengthening behavior caused by the misfit precipitate can be reflected by the dislocation slip geometry.

## 2. Materials and Methods

**Figure 1:**
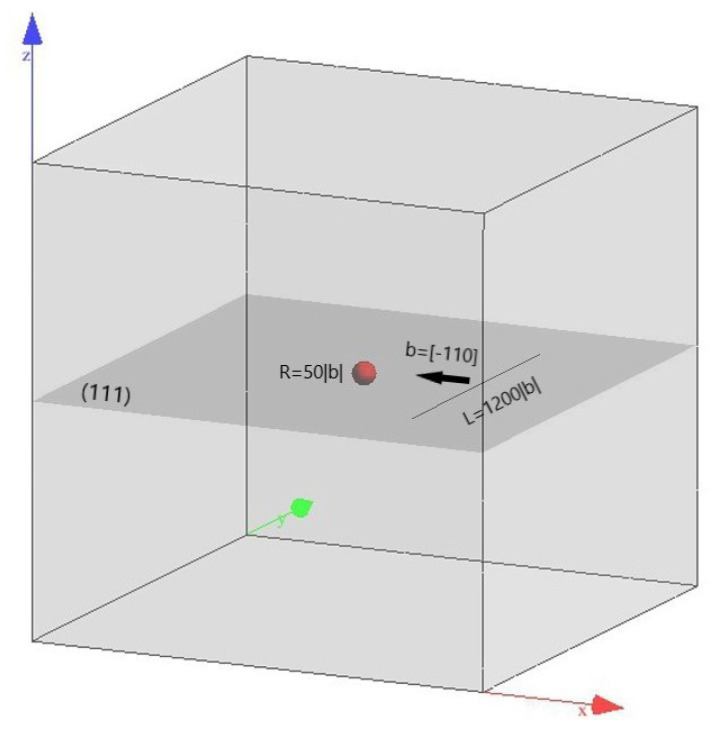
Schematics of the simulation model. The precipitate is represented by the red sphere, the dislocation line is represented by the black line. The light grey volume is the simulation volume, the dark grey plane is the (111) primary slip plane with z/R=0.

We simulate the interaction of a single dislocation line and one misfit spherical precipitate in this study. As shown in Figure 1, we assume the crystal face (111) and the crystal orientation [-110] as the primary slip system. Therefore we set the Cartesian axis as x = [1-10], y = [11-2], z = [111]. The origin of coordinates is located at the center of the spherical precipitate. An edge dislocation line (black line) with the Burgers vector b = [-110] and the initial tangent vector [0-10], lies along the y-axis at x=500|b| on the primary slip plane. The magnitude of the Burgers vector |b|=0.286 nm. With two pinned endpoints, the initial length of the dislocation line is 1200|b|. The precipitate (red sphere) with the radius R=50|b| is subjected to the dilatational eigenstrain ($\varepsilon_{ij}^{*}=\delta_{ij}\varepsilon_{0},\varepsilon_{0}=0.1$). The crystal properties of the matrix are the same as the aluminum, with the Young’s modulus E=70 GPa, the shear modulus μ=27 GPa, the Poisson’s ratio ν=0.3 and the lattice constant a=0.404 nm. The elastic modulus of the misfit precipitate is assumed to be the same as the matrix. Note that the dissimilar stiffness effect can also be analyzed by Eshelby’s inhomogeneity problem. However, when the equivalent inclusion with the fictitious eigenstrain is applied, there would be the same effect.

In our simulation model, the dislocation glide which is driven by the external stress has a constant strain rate ${\dot{\varepsilon }}_{13}=\varepsilon_{13}/∆t=10^{4}s^{-1}$ (∆t is the time step). Here is the relationship between the rate of external stress and the strain rate:

(1) $${\dot{\sigma }}_{13}=\mu \left({\dot{\varepsilon }}_{13}-{\dot{\varepsilon }}_{13}^{p}\right)$$

where μ is the shear modulus. Note that the dislocation velocity is proportionally changed with the force acting on the dislocation, the external stress level will be increased under a higher strain rate. The plastic strain rate ${\dot{\varepsilon }}_{13}^{p}$ is obtained from the motion of dislocation:

(2) $${\dot{\varepsilon }}_{13}^{p}=\frac{b∆A}{V∆t}$$

where ∆A is the swept area of the dislocation line, *V* is the volume of the matrix ($1.87\times 10^{-19}$ m^3^, ∆x=2000|b|,∆y=2000|b|,∆z=2000|b|). After obtaining the plastic strain through Equation (2), we could calculate the average value of the external stress by Equation (1).

We employ the parametric dislocation dynamics (PDD) to discretize the dislocation line into a series of curved segments. These segments are represented by the Burgers vector, the tangent vector, and the position vector at their endpoints. The length of the dislocation segments are ranged from 10|b| to 20|b|.

During the slip process, the dislocation segments experience the external stress $\sigma_{ij}^{0}$, the stress caused by the precipitate $\sigma_{ij}^{int\_par}$, the stress caused by itself and other dislocation segments $\sigma_{ij}^{int\_dis}$, as well as the friction shear stress caused by the crystal $\sigma_{ij}^{fr}$. For aluminum, $\sigma_{ij}^{fr}=3\times 10^{-5}\mu$, which can be neglected. So the total stress $\sigma_{ij}^{T}$ can be written as:

(3) $$\sigma_{ij}^{T}=\sigma_{ij}^{0}+\sigma_{ij}^{int\_par}+\sigma_{ij}^{int\_dis}$$

It is noted that to avoid the singularity, the stress caused by the adjoining segments is approximated by the line tension $F_{T}$,

(4) $$|F_{T}|=|\tau_{ij}b_{i}|=\frac{\alpha \mu b^{2}}{r}$$

where α=0.5, *r* is the local curvature of the dislocation segment. Meanwhile, the stress caused by the far-field finite segment can be expressed as follows:

(5) $$\sigma_{ij}=\frac{\mu b_{n}}{4\pi }\oint_{c}\left[\frac{1}{2}R_{,mpp}\left(\in_{jmn}dl_{i}+\in_{imn}dl_{j}\right)+\frac{1}{1-\nu }\in_{kmn}\left(R_{,ijm}-\delta_{ij}R_{,ppm}\right)dl_{k}\right]$$

where $R=x-x^{\prime }$, *x* is the field point, *x’* is the source point, $R_{,ijk}$ is the derivative of *R*, $\in_{ijk}$ is the permutation tensor, $\delta_{ij}$ is the Kronecker delta.

The stress caused by the precipitate will be introduced in the next section. With the stress tensor, we could calculate the Peach-Koehler force $f_{m}$ on the nodal points,

(6) $$f_{m}=\in_{jmn}\sigma_{ij}b_{i}\xi_{n}$$

where $\xi_{n}$ is the tangent vector of the dislocation nodes. Once we obtain the Peach–Koehler force, the nodal velocity $v_{i}$ can be calculated by the following equation,

(7) $$v{}_{i}=f{}_{g}\cdot M$$

$f_{g}$ is the shear component of Peach–Koehler force, *M* is the reciprocal of the dislocation drag coefficient, we assumed that $M=1.75\times 10^{4}$ (Pa· s)${}^{-1}$ in this model [18].

### 2.1. Stress Field of Spherical Precipitate

We assumed that the elastic modulus of the matrix and the misfit precipitate are the same, the material is elastically isotropic. According to Eshelby’s inclusion theory, the shear stress inside the precipitate is calculated to be 0. However, due to the dilatational eigenstrain, the stress exists outside around the precipitate. The internal stress outside the misfit spherical precipitate is calculated based on Green’s function proposed by Mura as Equation (8) [19],

(8) $$C_{jlmn}G_{ij,l}\left(\bar{x}\right)=\frac{-1}{8\pi (1-\nu )}\left[\left(1-2\nu \right)\frac{\delta_{mi}{\bar{x}}_{n}+\delta_{ni}{\bar{x}}_{m}-\delta_{mn}{\bar{x}}_{i}}{{\bar{x}}^{3}}+3\frac{{\bar{x}}_{m}{\bar{x}}_{n}{\bar{x}}_{i}}{{\bar{x}}^{5}}\right]$$

where $C_{jlmn}$ is the shear modulus, $G_{ij,l}$ is the derivative of Green’s function, $\bar{x}=\left|x-x^{\prime }\right|$, ${\bar{x}}_{i}=x_{i}-x_{i}^{\prime }$, ν is the Poisson’s ratio. The stress field can be calculated as follows,

(9) $$\sigma_{ij}=\frac{E}{1+\nu }\left[\left(\varepsilon_{ij}-\varepsilon_{ij}^{*}\right)+\delta_{ij}\frac{\nu }{1-2\nu }\left(\varepsilon_{kk}-\varepsilon_{kk}^{*}\right)\right]$$

where $\varepsilon_{ij}^{*}$ is the eigenstrain, $\varepsilon_{ij}$ is the strain field caused by the precipitate,

(10) $$\varepsilon_{ij}=-\frac{1}{2}\int_{|\Omega |}C_{klmn}\varepsilon_{mn}^{*}\left(x^{\prime }\right)\left[G_{ik,l}\left(x-x^{\prime }\right)n_{j}+G_{jk,l}\left(x-x^{\prime }\right)n_{i}\right]dS$$

where *n* is the normal vector of the integral surface *S*.

**Figure 2:**
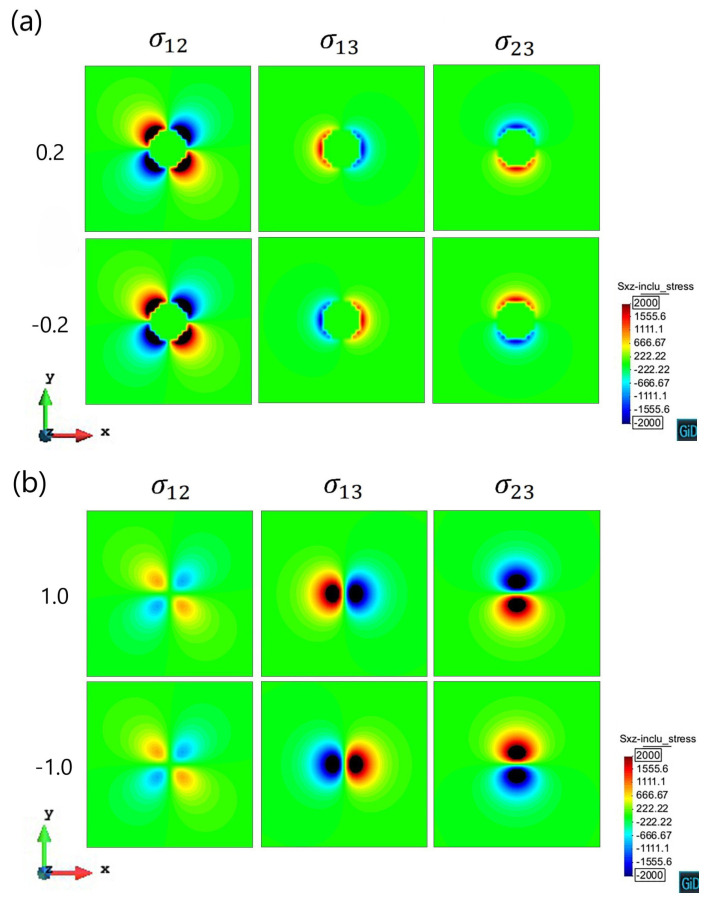
Contour plots of stress on the cross-section of the spherical precipitate (**a**) z/R=±0.2; (**b**) z/R=±1.0.

The computation results of the stress field caused by the spherical precipitate are shown in Figure 2, where the stress on the cross-section of the precipitate (at z/R=±0.2 and z/R=±1.0) is plotted as the contour plots. The magnitude range of the stress is from −2000 Pa to 2000 Pa. The shear stress inside the spherical precipitate is zero. For the edge dislocation segments, $\sigma_{12}$ and $\sigma_{23}$ have no contribution to the motion, $\sigma_{13}$ contributes to the glide motion. For the screw segments, $\sigma_{13}$ has no contribution to the motion, while $\sigma_{12}$ contributes to the cross-slip, $\sigma_{23}$ contributes to the glide and the double cross-slip motion.

As shown in the contour plots, when the sign of z/R inverses, the stress component $\sigma_{12}$ does not change, while the sign of $\sigma_{13}$ and $\sigma_{23}$ turn opposite. For cases z/R>0, the dislocation line with the tangent vector [0-10] expands along the -*x* direction on its slip plane, the precipitate stress component $\sigma_{13}$ is negative at the x>0 side and positive at the x<0 side. When the dislocation gets closer to the precipitate, the dislocation line suffers a repulsive force repelling it to approach the precipitate. After the dislocation bypassing the precipitate, it suffers a repulsive force to push it away from the precipitate. However, for the cases z/R<0, the situation turns opposite. In these cases, the dislocation line experiences an attractive force to approach the precipitate easier, and moves away from the precipitate harder. It is noted that the $\sigma_{13}$ on z/R=±1.0 planes is much larger than that on z/R=±0.2 planes, which means the dislocation line suffers a larger repulsive or attractive force on z/R=±1.0 planes than on z/R=±0.2 planes.

For the other stress components, the $\sigma_{12}$ on z/R=±0.2 planes is larger than that on z/R=±1.0 planes, which means the screw dislocation segments on z/R=±0.2 planes are easier to cross slip than on z/R=±1.0 planes. However, the $\sigma_{23}$ on z/R=±1.0 planes is larger than that on z/R=±0.2 planes, which means the screw segments on z/R=±1.0 planes are easier to double cross slip than on z/R=±0.2 planes.

### 2.2. The Cross-Slip Model and Annihilation Reaction

The cross-slip mechanism has a great influence on the dislocation bypassing process. While the dislocation topological change on the primary slip plane is independent of crystal structure (e.g., fcc and bcc), the cross-slip event would be influenced by the primary and secondary slip planes. In this model, we set the plane (11-1) as the secondary slip plane. Then define the direction of cross-slip as the cross product of the screw segment’s tangent direction and the secondary slip plane’s normal direction. Assume that the component of the Peach-Koehler force on the primary slip plane is $F_{G}$, while the component along the cross-slip direction is $F_{CS}$. The cross-slip happens whenever $F_{CS}>F_{G}$. If $F_{CS}\leq F_{G}$, the glide motion continues on the primary slip plane.

According to the definition of the screw dislocation, segment with the tangent direction ξ parallels to the Burgers vector *b* (|ξ×b|=0) is the pure screw segment. However, in our algorithm, the segment can be seen as a pure screw segment when |ξ×b|<0.1.

As for the annihilation reaction, it happens under the following two conditions: 1. The distance between two nodes is smaller than 20|b|. It is noted that we also insert a node at every dislocation segment’s midpoint; 2. The relationship between the tangent vector ξ and the Burgers vector *b* of nodes satisfies the following formulas,

$$\xi_{m}\cdot \xi_{n}\approx 1,b_{m}+b_{n}=0\;\mathrm{or}\;\xi_{m}\cdot \xi_{n}\approx -1,b_{m}-b_{n}=0$$

## 3. Results and Discussion

### 3.1. The Topological Evolution of the Dislocation around a Misfit Precipitate

In this section, we show the topological evolution of the dislocation line in z/R≥0 cases, then compared with z/R<0 cases. With different relative heights of the primary slip planes, the dislocation topological evolution varies.

**Figure 3:**
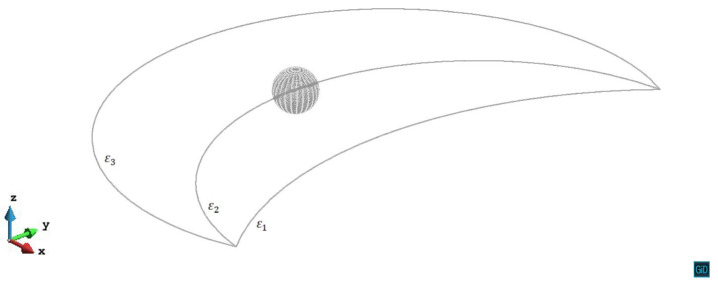
Process of the dislocation line with pinned endpoints passing a misfit spherical precipitate on the slip plane z/R=0. The plastic strain of different steps $\varepsilon_{1}=2.13\times 10^{-5}$, $\varepsilon_{2}=5.56\times 10^{-5}$, $\varepsilon_{3}=1.07\times 10^{-4}$.

The initial slip plane lies on the mid-plane of the precipitate (z/R=0). From Figure 3 we can see the process that the dislocation line gradually bows and passes the precipitate under the external stress without experiencing a repulsive force. The space between the dislocation line of different steps is the area in which the dislocation line swept during its glide motion. Because of the same shear modulus as the matrix and the symmetry of misfit spherical precipitate, the shear stress caused by the precipitate is zero on the z/R=0 slip plane. In view of the internal stress, z/R=0 case can be regarded as the condition without the precipitate.

**Figure 4:**
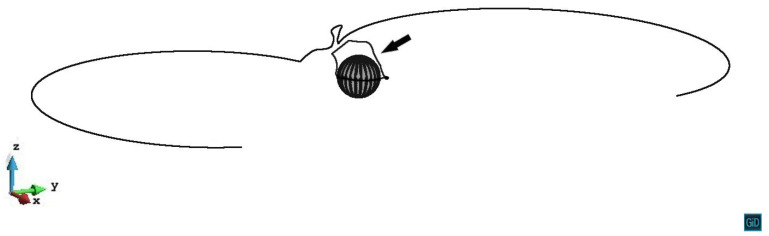
The dislocation line in z/R=0.2 case bypassing the precipitate with a prismatic loop (black arrow) left. The plastic strain $\varepsilon =1.69\times 10^{-4}$.

Figure 4 shows the primary slip plane z/R=0.2 case. As the dislocation line gets closer to the precipitate, the repulsive shear stress from the precipitate increases. The dislocation line bows around the precipitate to form the screw segments. The cross-slip happens on both sides of the precipitate and the screw segments cross slip up from the primary slip plane. In the stress field above the precipitate, the screw segments whose y>0 double cross slip along -*y* direction and approach the screw segments whose y<0. An annihilation reaction then occurs when the two parts of the screw segments are close enough to each other. Other parts of the dislocation reconnect then keep expanding. Finally, a prismatic loop (black arrow) is left after the dislocation line bypassing the precipitate. The video of z/R=0.2 case can be seen in the Supplemental Materials [S1]. Hatano [17] and Erel et al. [20] also reported a prismatic loop formed after the double cross-slip and the annihilation.

**Figure 5:**
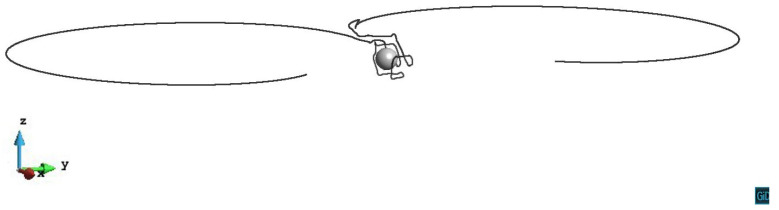
The dislocation line with the primary slip plane z/R=0.6 leaving a helix around the precipitate. The plastic strain $\varepsilon =6.19\times 10^{-4}$.

When the primary slip plane lies on z/R=0.6, the dislocation is pinned and bows around the precipitate firstly. Then the screw segments start to cross slip. For the screw segments whose x>0, they cross slip down from the original slip plane and double-cross slip to approach each other. Note that the annihilation of the dislocation in this model takes place whenever the dislocation segments are approached within the distance of 20|b|. The minimum distance between the dislocation segments observed in Figure 5 is 57.8|b|, therefore the annihilation does not happen in this case. For the screw segments with x<0, they cross slip up from the primary slip plane and double-cross slip along the *y* axis to the other side of the precipitate. However, the annihilation reaction does not occur either. After these two parts of the screw segments entering another side of the precipitate stress field, they start to cross slip down from the double-cross slip plane. Finally, the dislocation line bypasses the precipitate by creating a helix around it. The video of z/R=0.6 case can be seen in the Supplemental Materials [S3].

**Figure 6:**
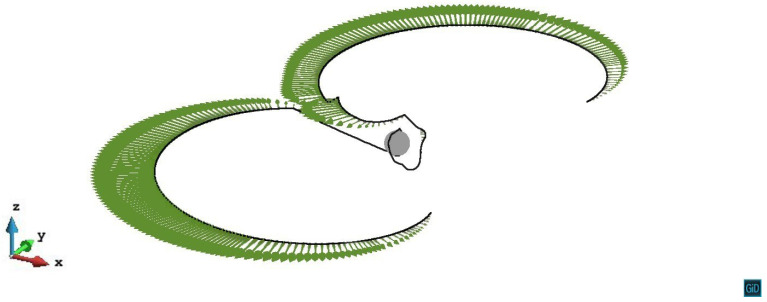
The dislocation topology and nodal velocity vectors of the dislocation line with the primary slip plane z/R=1.0. The plastic strain $\varepsilon =2.69\times 10^{-4}$.

For the primary slip plane z/R=1.0, Figure 6 shows the nodal velocity vectors of the dislocation line. We can see that the dislocation also creates a helix around the precipitate. The dislocation segments far from the precipitate still have the trend to expand, while the segments near the precipitate are almost pinned and can hardly move. For the left side of the bowing dislocation, we can see that it connects a long straight dislocation segment without velocity. According to $\sigma_{23}$ as shown in Figure 2, the force balance is satisfied by the screw segments along the *x*-axis, which leads to the formation of the straight dislocation line. The video of z/R=1.0 case can be seen in the Supplemental Materials [S5].

**Figure 7:**
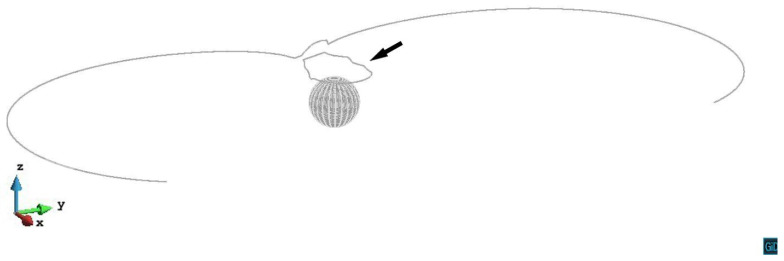
The dislocation line in z/R=1.4 case bypassing the precipitate with an Orowan loop (black arrow) left. The plastic strain $\varepsilon =3.06\times 10^{-4}$.

The dislocation evolution in z/R=1.4 case is shown in Figure 7. The stress from the precipitate is large enough to pin the dislocation segments above the precipitate, but not enough to trigger a large-scale cross-slip, only slight cross-slip happens. Finally, an Orowan loop (black arrow) is left above the precipitate after the dislocation line bypassing the precipitate stress field. The video of z/R=1.4 case can be seen in the Supplemental Materials [S7].

**Figure 8:**
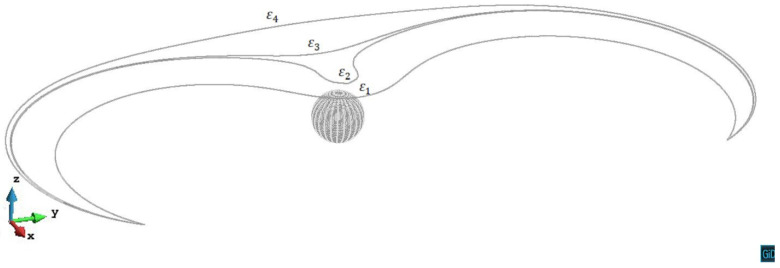
Process of the dislocation line gradually unpins from the precipitate stress field on the plane z/R=1.8. The plastic strain of different steps $\varepsilon_{1}=7.77\times 10^{-5}$, $\varepsilon_{2}=7.85\times 10^{-5}$, $\varepsilon_{3}=7.86\times 10^{-5}$, $\varepsilon_{4}=7.93\times 10^{-5}$.

In z/R=1.8 and z/R=2.2 cases, the internal stress from the precipitate is relatively weak, therefore can neither pin the dislocation segments nor trigger the cross-slip. In Figure 8, we can see the process that the dislocation line on the slip plane z/R=1.8 first being retarded by the precipitate stress field, then gradually unpins under the increased external stress. The situation on the z/R=2.2 plane is almost the same as on the z/R=1.8 plane.

**Figure 9:**
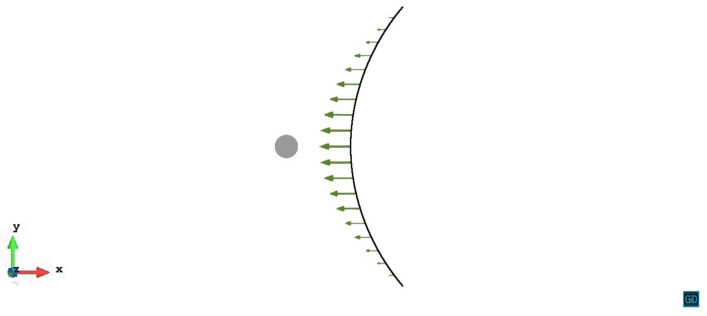
The dislocation topology and nodal force vectors in z/R=−0.2 case. The plastic strain $\varepsilon =2.23\times 10^{-5}$.

**Figure 10:**
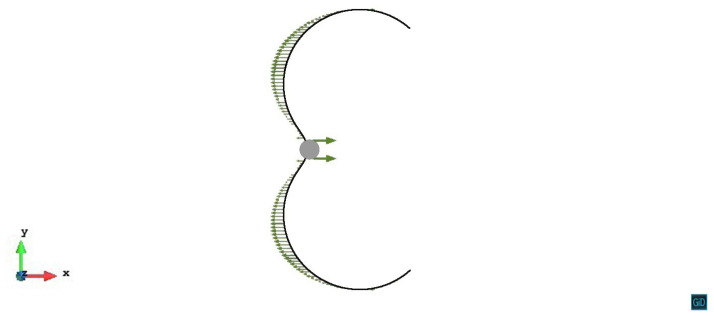
The dislocation topology and nodal force vectors in z/R=−0.2 case. The plastic strain $\varepsilon =9.41\times 10^{-5}$.

In z/R<0 cases, as predicted in Section 2.1, the stress tensor $\sigma_{13}$ reverses. So the repulsive force between the precipitate and the dislocation turns to an attractive force. For the primary slip plane z/R=−0.2, we visualized the nodal force vectors in the following two figures. From Figure 9, we can see that under external stress and the precipitate internal stress, the dislocation line is attracted to the precipitate. In Figure 10, the dislocation line cuts into the precipitate and bows around it. The dislocation topological evolution corresponds with the nodal force vectors.

**Figure 11:**
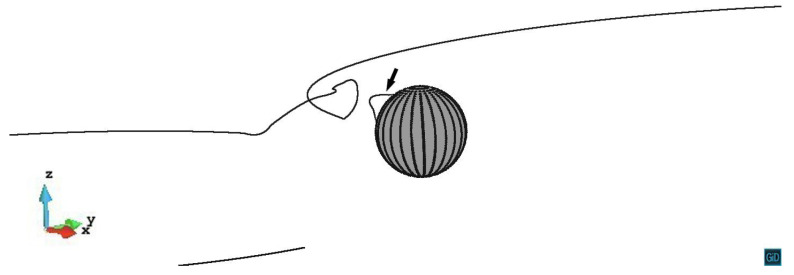
The dislocation line in z/R=−0.2 case bypassing the precipitate with a prismatic loop (black arrow) left. The plastic strain $\varepsilon =2.31\times 10^{-4}$.

As shown in Figure 11, before leaving the precipitate, the cross-slip and an annihilation reaction happen. A prismatic loop (black arrow) is left behind after the dislocation line bypassing the precipitate. The video of z/R=−0.2 case can be seen in the Supplemental Materials [S2].

**Figure 12:**
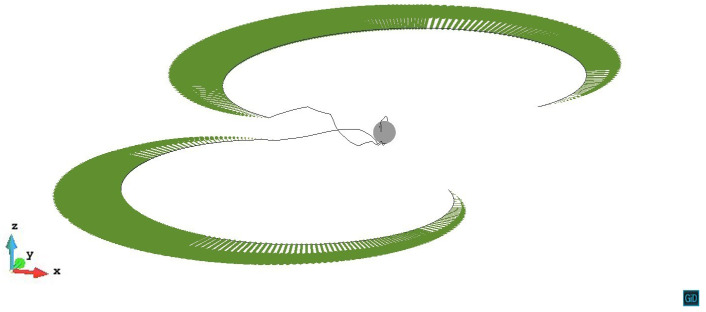
The dislocation topology and nodal velocity vectors in z/R=−0.6 case. The plastic strain $\varepsilon =4.68\times 10^{-4}$.

**Figure 13:**
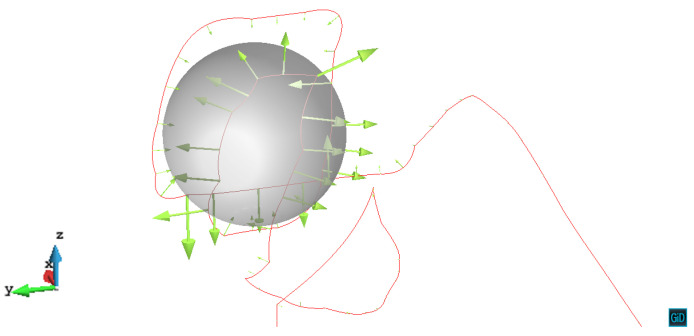
The dislocation nodal PK force vectors around the precipitate in z/R=−0.6 case.

In z/R=−0.6 case, the dislocation line cuts into the precipitate and bows around it, then the cross-slip happens. The dislocation line creates a helix around the precipitate to bypass it. Figure 12 shows the nodal velocity vectors of the dislocation. We can see that the dislocation nodes near the precipitate have no velocity. As shown in Figure 13, inside the spherical precipitate, the magnitude of PK force is large due to the normal stress component. The force vectors’ direction of the inside nodes point outward of the precipitate, which are opposite to the nodes located outside the precipitate. The video of z/R=−0.6 case can be seen in the Supplemental Materials [S4].

**Figure 14:**
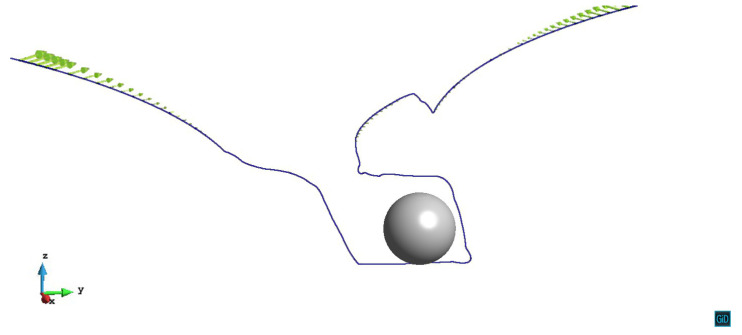
The dislocation topology and nodal velocity vectors in z/R=−1.0 case. The plastic strain $\varepsilon =9.64\times 10^{-4}$.

In z/R=−1.0 case as shown in Figure 14, after the dislocation line bowing around the precipitate, both sides of the screw segments cross slip up from the primary slip plane. Then the screw segments whose y>0,z>0 double cross slip along the -*y* direction and come closer to the segments whose y<0. However, the annihilation conditions are not satisfied because the minimum distance of the dislocation segments observed in this figure is 61.6|b|. Finally, a partial prismatic loop formed around the precipitate. The video of z/R=−1.0 case can be seen in the Supplemental Materials [S6].

The similar topological evolution as z/R=−1.0 case happens in z/R=−1.4 case. After the double cross-slip, the screw segments whose y>0,z>0 slightly cross slip down from the double cross-slip plane, but the annihilation conditions are still not satisfied, because the minimum distance between two screw segments is 31.8|b|. Finally, a partial prismatic loop formed again.

**Figure 15:**
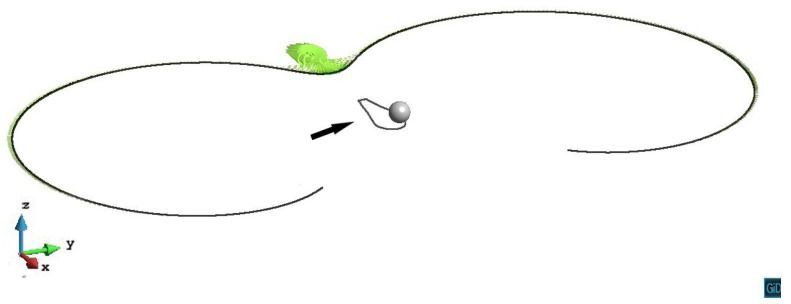
The dislocation nodal velocity vectors and an Orowan loop (black arrow) is left in z/R=−1.8 case. The plastic strain $\varepsilon =6.34\times 10^{-4}$.

In z/R=−1.8 case, the cross-slip does not happen. As shown in Figure 15, both sides of the screw segments get closer to each other on the primary slip plane, then annihilate to form an Orowan loop (black arrow). The other parts of the dislocation line keep expanding. For the middle part of the dislocation line, the nodes have the larger velocity vectors due to the larger line tension acting on these nodes.

**Figure 16:**
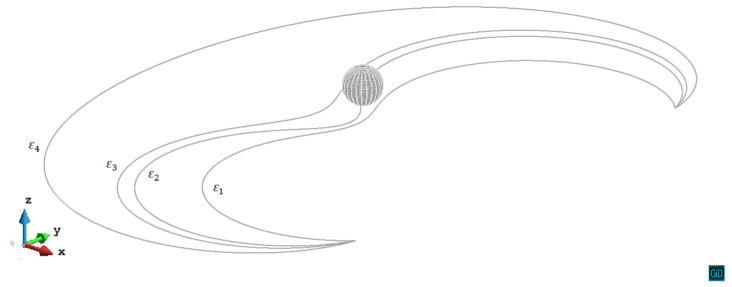
The gradual unpinning process of the dislocation line on the z/R=−2.2 plane. The plastic strain of different steps $\varepsilon_{1}=9.30\times 10^{-5}$, $\varepsilon_{2}=1.28\times 10^{-4}$, $\varepsilon_{3}=1.43\times 10^{-4}$, $\varepsilon_{4}=2.24\times 10^{-4}$.

According to Figure 16, in z/R=−2.2 case, the dislocation line being retarded by the precipitate stress field and bows on the primary slip plane. As the internal stress from the precipitate is relatively weak on this plane, the cross-slip does not happen and the dislocation line gradually unpins under the increased external stress.

After comparing the dislocation topological evolution of z/R>0 cases to z/R<0 cases, we could point out two main differences. First is that due to the inverse sign of $\sigma_{13}$, the internal shear stress acting on the edge dislocation segments is a repulsive force in z/R>0 cases, while it is an attractive force in z/R<0 cases. The dislocation line glides fast on its primary slip plane towards the precipitate in z/R<0 cases. While in z/R>0 cases, the repulsive shear stress from the precipitate makes the dislocation line glide much slower than on z/R<0 planes. The second is that in 0<z/R<1.0 cases, the repulsive shear force makes the dislocation line bow around the precipitate, while in −1.0<z/R<0 cases, the attractive shear force attracts the dislocation line to cut into the precipitate.

### 3.2. The Stress and Energy Analysis of Different Slip Planes

#### 3.2.1. Energies Associated with the Dislocation Motion

In this model, the Gibbs free energy $E_{G}$ consists of the elastic strain energy $E_{el}$ and the potential energy *P*. While the elastic energy $E_{el}$ contains the matrix intrinsic elastic energy $E_{el}^{0}$, the precipitate self-energy $E_{el}^{par}$, the dislocation self-energy $E_{el}^{dis}$ and the interaction energy between the precipitate and the dislocation $E_{el}^{par\_dis}$. It is noted that $E_{el}^{dis}$ contains the dislocation self-energy and the interaction energy between the dislocation segments. We assume that *D* as the matrix and Ω as the precipitate.

(11) $$E_{G}=E_{el}+P=E_{el}^{0}+E_{el}^{par}+E_{el}^{dis}+E_{el}^{par\_dis}+P$$

the elastic strain energy $E_{el}$ can be written as

(12) $$\begin{array}{cc} E_{el}= & \frac{1}{2}\int_{D}\sigma_{ij}^{0}e_{ij}^{0}dV+\frac{1}{2}\int_{D}\sigma_{ij}^{par}e_{ij}^{par}dV+\frac{1}{2}\int_{D}\sigma_{ij}^{dis}e_{ij}^{dis}dV+\\ & \int_{D}\sigma_{ij}^{par}e_{ij}^{dis}dV \end{array}$$

where $\sigma_{ij}^{par}$ is the stress tensor of the precipitate, $\sigma_{ij}^{dis}$ is the stress tensor of the dislocation, $e_{ij}^{0}$ is the elastic strain of the matrix, $e_{ij}^{par}$ is the elastic strain of the precipitate, $e_{ij}^{dis}$ is the elastic strain of the dislocation.

Note that the fourth term of Equation (12) is the interaction energy between the precipitate and the dislocation line. The positive interaction energy indicates the precipitate retards the dislocation motion, while the negative interaction energy indicates that the precipitate promotes the dislocation motion or makes the dislocation segments cross slip. So the interaction energy can be used to measure the level of precipitate strengthening. Since $e_{ij}=\varepsilon_{ij}-\varepsilon_{ij}^{*}$, $\varepsilon_{ij}$ is the total strain, $\varepsilon_{ij}^{*}$ is the eigenstrain. Equation (12) can be rewritten as:

(13) $$\begin{array}{cc} E_{el}= & \frac{1}{2}\int_{D}\sigma_{ij}^{0}e_{ij}^{0}dV+\frac{1}{2}\int_{D}\sigma_{ij}^{par}\left(\varepsilon_{ij}^{par}-\varepsilon_{ij}^{*par}\right)dV+\frac{1}{2}\int_{D}\sigma_{ij}^{dis}\left(\varepsilon_{ij}^{dis}-\varepsilon_{ij}^{*dis}\right)dV+\\ & \int_{D}\sigma_{ij}^{par}\left(\varepsilon_{ij}^{dis}-\varepsilon_{ij}^{*dis}\right)dV \end{array}$$

where $\varepsilon_{ij}^{*par}$ is the eigenstrain of precipitate, $\varepsilon_{ij}^{*dis}$ is the eigenstrain in the matrix *D* caused by the dislocation. The free surface condition is maintained by the internal stress ($\sigma_{ij}n_{j}=0$) and the equilibrium condition ($\sigma_{ij,j}=0$) inside the matrix *D*. Equation (13) can be reduced as follows,

(14) $$E_{el}=\frac{1}{2}\int_{D}\sigma_{ij}^{0}e_{ij}^{0}dV-\frac{1}{2}\left(\int_{\Omega }\sigma_{ij}^{par}\varepsilon_{ij}^{*par}dV+\int_{D}\sigma_{ij}^{dis}\varepsilon_{ij}^{*dis}dV+2\int_{D}\sigma_{ij}^{par}\varepsilon_{ij}^{*dis}dV\right)$$

The potential energy *P*, which is obtained from the following Equation (15), equals the total work done by the external stress,

(15) $$P=-W=-\int_{S}F_{i}\left(u_{i}^{0}+u_{i}^{par}+u_{i}^{dis}\right)dS$$

where the surface traction $F_{i}=\sigma_{ij}^{0}n_{j}$, the displacement of surface S consists of $u_{i}^{0}$, $u_{i}^{par}$ and $u_{i}^{dis}$, which are the displacement caused by the surface traction, the precipitate and the dislocation respectively.

The dissipated energy $E_{D}$ is calculated by the following Equation (16), equals the plastic work done by the external stress,

(16) $$E_{D}=-\int_{S}F_{i}\cdot u_{i}^{dis}dS$$

As the dislocation motion during a time step is realized under a constant applied stress in this model, the stored energy $E_{S}$ can be assumed as follows,

(17) $$E_{S}=∆E_{el}^{dis}+∆E_{el}^{par\_dis}$$

where $∆E_{el}^{dis}$ is the increasing magnitude of the dislocation self-energy and $∆E_{el}^{par\_dis}$ is the change of the precipitate-dislocation interaction energy.

#### 3.2.2. Analysis of the Stress and Energy Curves

In this part, we plotted and analyzed the stress–strain curve as well as the energy-strain curves on different heights of the primary slip plane. To find out the influence of dislocation evolution on stress, energies, and the strengthening mechanism.

**Figure 17:**
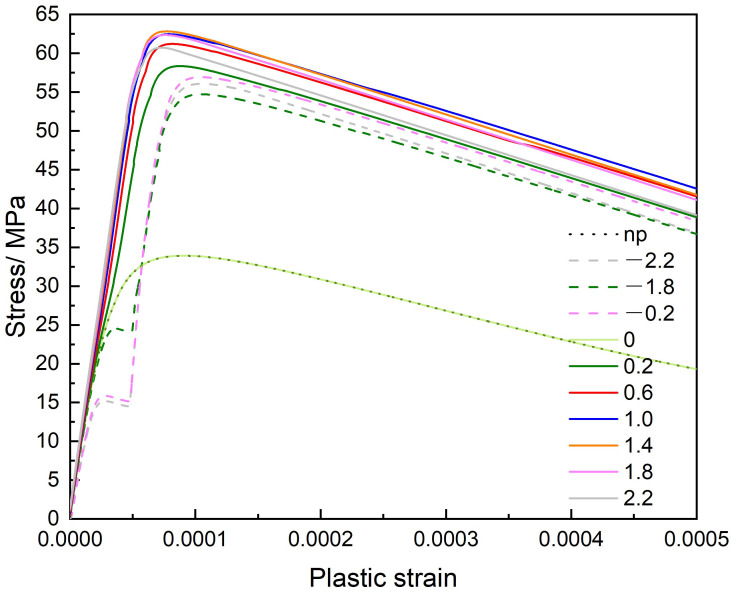
The stress–strain curve.

Figure 17 is the stress–strain curve, relative heights between the primary slip plane and the mid-plane (z/R=0) vary from z/R=−2.2 to z/R=2.2. The dislocation interacting with a single precipitate is considered, the stress level is much increased by the existing precipitate as compared with the stress–strain curve without the precipitate (as indicated by np). We can see that the stress curve of z/R=0 case is coincident with that no precipitate (np) case. Which means the internal stress from the precipitate does not influence the dislocation motion on the z/R=0 plane. When the dislocation slip plane is above the mid-plane (z/R>0), the hardening behavior is obvious on the stress–strain curve, which implies the dislocation is retarded by the precipitate. When the dislocation slip plane is below the mid-plane (z/R<0), we can see that the stress is released when the strain is around 0.00003−0.00004 because while the precipitate attracts the dislocation line to approach it, the external stress decreases to maintain the constant strain rate. Due to the opposite sign of $\sigma_{13}$, the maximum stress values on z/R>0 planes are greater than that on z/R<0 planes, which indicates the stronger strengthening effect on the z/R>0 slip planes. The strengthening effect exists in each case except on the mid-plane (z/R=0) of the spherical misfit precipitate. Theoretically, the dislocation pinning stress level can be derived directly from the Orowan stress, which is determined by the initial structure of the present model. However, the stress–strain curve indicates that the obstacle with the misfit eigenstrain can influence the pinning stress level, depending on the relative heights of the primary slip plane.

**Figure 18:**
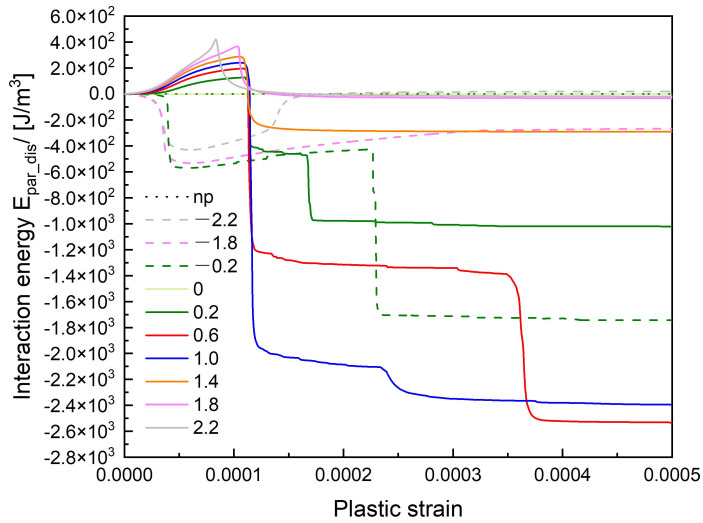
The interaction energy curve between the dislocation and precipitate.

**Figure 19:**
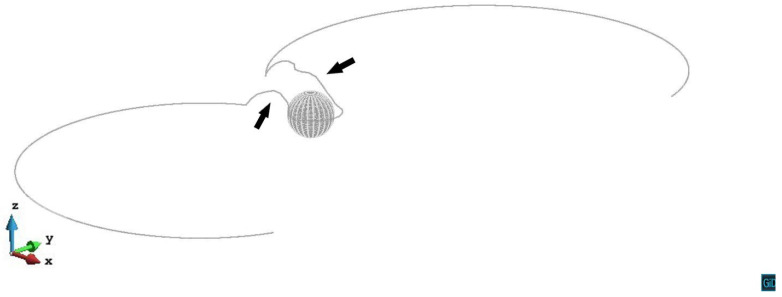
Snapshot of the dislocation cross-slip (black arrows) on z/R=0.2 plane. The plastic strain $\varepsilon =1.46\times 10^{-4}$.

The interaction energy curve between the dislocation and the precipitate shown in Figure 18 reflects the details of the dislocation motion. On the z/R=0 mid-plane, the internal stress due to the precipitate does not influence the dislocation motion, so the interaction energy remains zero, the dislocation gradually passes the precipitate under the external stress without retard. On the planes above the mid-plane (z/R>0), as the dislocation glide motion being retarded by the precipitate, the interaction energy gradually increases until the $\varepsilon_{p}=0.0001$. In z/R=0.2, z/R=0.6, and z/R=1.0 cases, the stress field of the precipitate makes the dislocation segments cross slip (black arrows in Figure 19) to bypass the precipitate, which largely decreases the interaction energy. There is a second step on these three interaction energy curves respectively because more dislocation segments cross slip at that stage. On the z/R=1.4 slip plane, the dislocation avoids sweeping the whole retarding area by forming an Orowan loop, which leads to the reduction of the interaction energy. The cross-slip slightly happens on the z/R=1.4 plane, results in more reduction of the interaction energy than in z/R=1.8 case.

**Figure 20:**
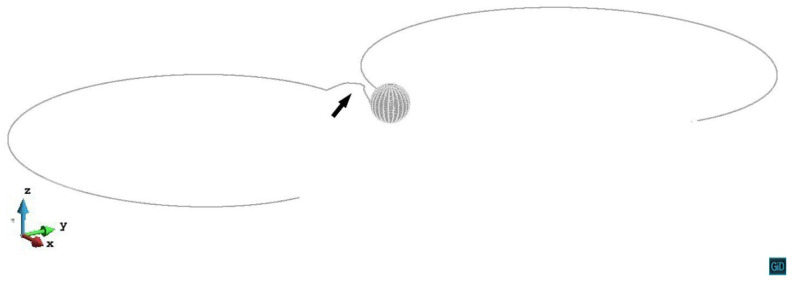
Snapshot of the dislocation cross-slip (black arrow) on the z/R=−0.2 plane. The plastic strain $\varepsilon =2.28\times 10^{-4}$.

In z/R<0 cases, the stress field of the precipitate attracts the dislocation approaching it, the interaction energy goes down at first. During the pinning process, the interaction energy increases. For z/R=−0.2 case, the cross-slip happens (black arrow in Figure 20) and the interaction energy suddenly drops when the strain is around 0.00023. For z/R=−1.8 and −2.2 cases, there is no cross-slip happens, the precipitate keeps doing positive work to the dislocation line, which results in the increase of the interaction energy. It is interesting to see that the interaction energy is eventually zero in z/R=2.2 and −2.2 cases, their curves show an increase and decrease of the interaction energy at first, but the energy is conserved after the dislocation bypassing the precipitate, this is due to the dislocation motion on the slip plane without the cross-slip. In z/R=−1.8 case, the energy conservation is not satisfied, the value of the interaction energy is lower than zero because of the Orowan loop formation. This fact also reflects the relaxation of the internal stress around the precipitate by the dislocation loop formation.

**Figure 21:**
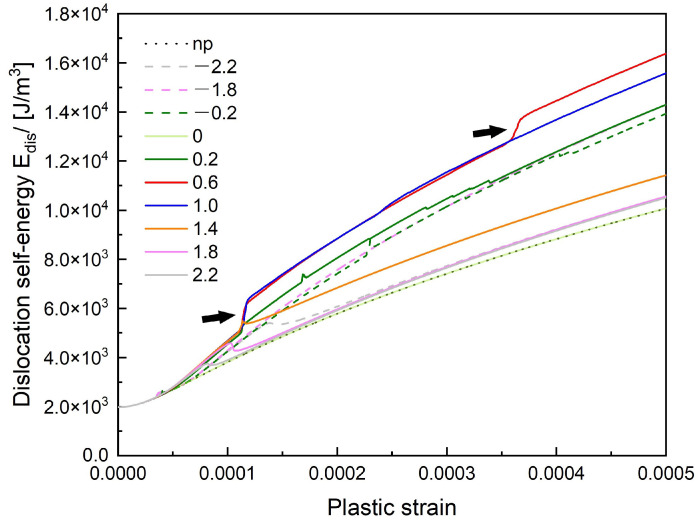
The dislocation self-energy curve. The increase steps in z/R=0.6 and z/R=1.0 cases due to a large-scale cross-slip are pointed by black arrows.

Figure 21 is the dislocation self-energy curve. When the cross-slip happens, the density of the dislocation increases, which results in the increase of the dislocation self-energy. On the contrary, after the annihilation reaction, some dislocation segments would return from the cross-slip plane to the primary slip plane, which leads to a slightly decrease of the dislocation self-energy. For z/R=0.6 and 1.0 cases, a large-scale cross-slip happens and there is no annihilation reaction, we can see huge increase steps (black arrows) on their curves. In z/R=−0.2 case, the cross-slip and the annihilation reaction result in the small zig-zag steps on its curve.

**Figure 22:**
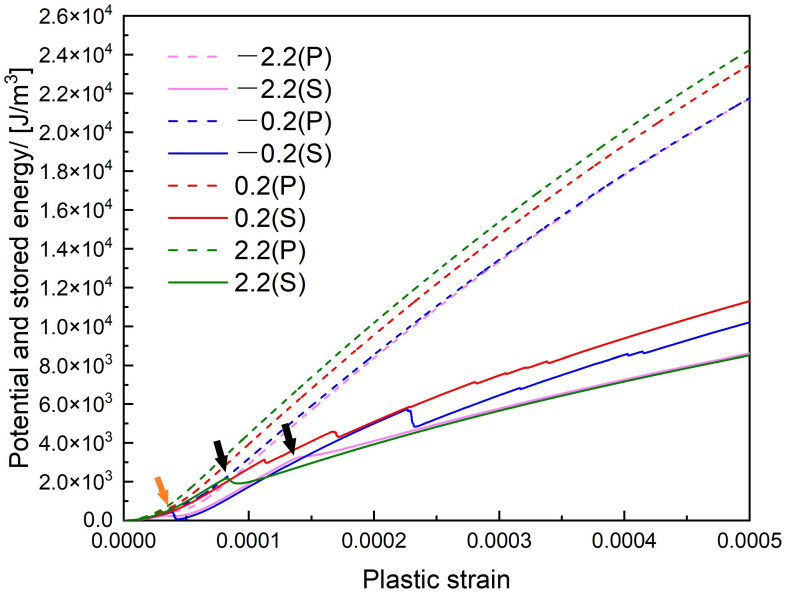
The potential and stored energy curve. The decrease steps in z/R=−0.2 and z/R=−2.2 cases due to an attractive interaction force are pointed by the orange arrow, the gentle decrease in z/R=2.2 and z/R=−2.2 cases due to the gradual unpinning process are pointed by the black arrows.

The potential energy contains the dissipated energy and the stored energy. According to Equation (15), the potential energy has the same tendency as the external stress. As shown by dotted lines in Figure 22, the potential energy is larger in z/R>0 cases than that in z/R<0 cases due to the larger external stress.

The stored energy which includes the dislocation self-energy and the interaction energy, can reflect the strong or weak hardening behavior. The decrease steps can be seen in z/R=0.2 and −0.2 cases when the cross-slip happens, this fact also indicates that the cross-slip event driven by the internal stress of the precipitate is a stress relaxation process. After the annihilation reaction in z/R=0.2R and −0.2R cases, some of screw segments cross slip back to the primary slip plane makes the dislocation density decrease, therefore we could also see a slight decrease in their stored energy curves. Note that the first decrease (orange arrow) in z/R=−0.2 and −2.2 cases at ε=0.00003 is caused by the decrease of the interaction energy since the precipitate attracts the dislocation line at that time. In z/R=2.2 and −2.2 cases, the cross-slip does not happen and we can see a gentle decrease (black arrows) on their stored energy curves, which is caused by the decrease of the dislocation self-energy during the gradual unpinning process. The general trend of the stored energy curve is increasing, because the bowing out of the dislocation line leads to the increase of the dislocation density and the self-energy.

The present model analyzed the influence of spherical misfit precipitate on the pinning stress level of the dislocation. The strengthening level of the pinning stress is quite increased by the misfit stress of the precipitate when the dislocation bypassing. The strong or weak hardening behavior can be reflected through the stored energy curve. When the cross-slip happens, the decrease of the interaction energy is larger than the increase of the dislocation self-energy, which leads to the decrease in the stored energy and the strengthening level. While during other times, the increasing trend of the stored energy corresponds with the dislocation self-energy, which indicates the increasing strengthening level during times without the cross-slip.

## 4. Conclusions

In this study, we simulated a single dislocation line interacting with one misfit spherical precipitate on different heights of the primary slip plane. The simulation is conducted with the parametric dislocation dynamics (PDD) method based on Green’s function. The cross-slip mechanism and the annihilation reaction are considered, the dislocation topological evolution differs on the different relative heights (z/R) of the primary slip plane, the simulation results show that three kinds of dislocation topological evolution occur: loop-forming (Orowan loop or prismatic loop),helix-forming, and gradual unpinning. The internal stress is deduced from Eshelby’s inclusion theory and Green’s function. When the sign of relative height z/R inverses, the stress components $\sigma_{13}$ and $\sigma_{23}$ also inverse. In z/R>0 cases, the interaction force between the precipitate and the dislocation is a repulsive one, while in z/R<0 cases it is an attractive one. The present model analyzed the influence of the spherical misfit precipitate on the dislocation pinning stress level. Theoretically, the dislocation pinning stress level can be derived directly from the Orowan stress, which is determined by the initial structure of the present model. However, our research results indicate that the obstacle with misfit eigenstrain can influence the pinning stress level, depending on the relative heights of the primary slip plane. The strengthening level of the pinning stress is much increased by the misfit stress of the precipitate when the dislocation bypassing. The strong or weak hardening behavior can be reflected through the stored energy curve. When the cross-slip happens, the decrease of the interaction energy is larger than the increase of the dislocation self-energy, which leads to the decrease in the stored energy and the strengthening level. During other times, the increasing trend of the stored energy corresponds with the dislocation self-energy, which indicates the increasing strengthening level during times without the cross-slip.

## Data Availability

Not applicable.

## Supplementary Materials

The following are available at https://www.mdpi.com/article/10.3390/ma14216368/s1, animation files of the dislocation topological evolution in video S1: z/R=0.2 case, video S2: z/R=−0.2 case, video S3: z/R=0.6 case, video S4: z/R=−0.6 case, video S5: z/R=1.0 case, video S6: z/R=−1.0 case, video S7: z/R=1.4 case.
